# Resting-State Brain Signal Variability in Prefrontal Cortex Is Associated With ADHD Symptom Severity in Children

**DOI:** 10.3389/fnhum.2018.00090

**Published:** 2018-03-12

**Authors:** Jason S. Nomi, Elana Schettini, Willa Voorhies, Taylor S. Bolt, Aaron S. Heller, Lucina Q. Uddin

**Affiliations:** ^1^Department of Psychology, University of Miami, Coral Gables, FL, United States; ^2^Department of Psychiatry and Human Behavior, Brown University, Providence, RI, United States; ^3^Neuroscience Program, University of Miami Miller School of Medicine, Miami, FL, United States

**Keywords:** ADHD, brain signal variability, resting-state fMRI, MSSD, prefrontal cortex

## Abstract

Atypical brain function in attention-deficit/hyperactivity disorder (ADHD) has been identified using both task-activation and functional connectivity fMRI approaches. Recent work highlights the potential for another measure derived from functional neuroimaging data, brain signal variability, to reveal insights into clinical conditions. Higher brain signal variability has previously been linked with optimal behavioral performance. At present, little is known regarding the relationship between resting-state brain signal variability and ADHD symptom severity. The current study examined the relationship between a measure of moment-to-moment brain signal variability called mean-square successive difference (MSSD) and ADHD symptomatology in a group of children (7–12 years old) with (*n* = 40) and without (*n* = 30) a formal diagnosis of ADHD. A categorical analysis comparing subjects with and without a clinical diagnosis of ADHD showed no differences in MSSD between groups. A dimensional analysis revealed a positive relationship between MSSD and overall ADHD symptom severity and inattention across children with and without an ADHD diagnosis. Specifically, this positive relationship was found in medial prefrontal areas comprising the default mode network. These results demonstrate a link between intrinsic brain signal variability and ADHD symptom severity that cuts across diagnostic categories, and point to a locus of dysfunction consistent with previous neuroimaging literature.

## Introduction

Attention-deficit/hyperactivity disorder (ADHD) is characterized by a lack of attention, impulsivity, and hyperactivity and affects ~11% of young individuals aged 4–17 years old (Visser et al., 2014). Contemporary theories posit that beyond dysfunction of prefrontal-striatal circuitry, ADHD symptomatology can also be linked to atypical patterns of functional connectivity within and among a number of large-scale brain networks (Castellanos and Proal, 2012). While a great deal of recent work has focused on delineating this atypical neural circuitry (Castellanos and Aoki, 2016), other neural properties associated with the disorder remain relatively unexplored.

Recently, analysis of BOLD signal variability has emerged as a valuable tool for investigating individual differences in behavioral performance. Increased BOLD signal variability during fixation periods in a task-based fMRI paradigm is more prevalent in younger compared with older adults (Garrett et al., 2010). These increases in BOLD signal variability were found in frontal, parietal, temporal, and subcortical brain areas. Intrinsic BOLD signal variability in resting-state fMRI data has been shown to generally decrease for most areas of the brain across the lifespan and increase within the insula and ventral temporal cortex (Nomi et al., 2017). These studies demonstrate how intrinsic BOLD signal variability measures can identify meaningful differences between and across subject populations.

Previous electrophysiological (Alba et al., 2016) and task-based fMRI (Depue et al., 2010) investigations have identified increased brain signal variability in participants with ADHD. However, there has been virtually no work exploring how underlying intrinsic brain signal variability is related to ADHD symptomatology using resting-state fMRI approaches. Investigations of variability in individuals with ADHD have traditionally focused on behavioral measures of reaction time or electrophysiological activity. Behavioral studies have demonstrated that individuals with ADHD generally exhibit greater reaction time variability that is thought to arise from momentary lapses in attention (Alderson et al., 2007). Electrophysiological investigations have shown that individuals with ADHD have increased variability of intrinsic functional connections between frontal, parietal, occipital, and temporal brain areas (Barttfeld et al., 2014; Alba et al., 2016).

We are currently aware of only two studies that have investigated brain signal variability in ADHD using fMRI (Depue et al., 2010; Sørensen et al., 2016). Depue and colleagues demonstrated that BOLD variability during an executive function Stroop task was higher in young adults with ADHD in the ventral medial prefrontal cortex (MPFC), parietal, subcortical, and cerebellar brain areas compared with young adults without ADHD (Depue et al., 2010). Sørensen and colleagues examined trial-by-trial variability during an odditory oddball paradigm and showed that adolescents with ADHD had greater BOLD amplitude variabilty compared with adolescents without ADHD. Higher BOLD amplitude variability in individuals with ADHD was found in the ventral MPFC as well as the basal ganglia (Sørensen et al., 2016). However, after controlling for age and IQ, only amplitude variability in the ventral MPFC remained significantly higher in adolescents with ADHD compared with adolescents without ADHD. Both of these studies demonstrate that individuals with ADHD have greater BOLD signal variability compared with indivduals without ADHD during task performance.

It is currently unknown if resting-state brain signal variability is greater in individuals with ADHD compared with individuals without ADHD. Previous fMRI studies have demonstrated that the intrinsic functional organization of the brain during rest is similar to the functional organization of the brain during task-states (Cole et al., 2014; Bolt et al., 2017). Additionally, the functional organization of the brain during rest can predict the functional organization of the brain during task-based fMRI (Cole et al., 2016; Tavor et al., 2016). These studies support the idea that the increased fMRI resting-state brain signal variability in individuals with ADHD may underlie the increased brain signal variability found during task performance.

The current study aimed to identify categorical and dimensional relationships between BOLD brain signal variability and ADHD symptom severity in children using resting-state fMRI data. We computed a whole-brain voxel-wise measure of BOLD signal variability called mean-square successive difference (MSSD) previously used in task-based (Samanez-Larkin et al., 2010) and resting-state (Nomi et al., 2017) fMRI investigations to identify categorical and dimensional relationships between MSSD and ADHD symptomatology. A categorical analysis compared MSSD between individuals with and without a diagnosis of ADHD, while a dimensional analysis examined the relationship between MSSD and ADHD symptom severity scores across individuals with and without an ADHD diagnosis. Based on task-based fMRI findings in individuals with ADHD (Depue et al., 2010; Sørensen et al., 2016), we hypothesized that increased resting-state brain signal variability would be associated with a diagnosis of ADHD and greater ADHD symptom severity.

## Methods

### Participants

Data for 222 participants were downloaded from the ADHD-200 database (http://fcon_1000.projects.nitrc.org/indi/adhd200/) (Milham et al., 2012). All participants were from the New York University Child Study Center at the Langone Medical Center. As the goal of the current study was to investigate young children with ADHD, individuals over the age of 12 were not included (88 participants). From the remaining 134 participants, some participants had two resting state scans for a total of 200 scans; for these individuals, we selected the resting state scan with lower levels of motion. Any scan with high absolute motion (>3 mm absolute rotation and/or translation) was removed (37 scans; 15 participants) and any participant with low IQ (Full-IQ < 80) or no IQ scores were also removed (four participants). Participants with negative handedness scores or no handedness scores were also removed (four participants). The remaining scans were used to match participants using random selection across groups on IQ, age, framewise displacement (FD), and the total number of volumes with an FD less than 0.5 mm (*p* > 0.1) (Table 1). The final dataset included 70 participants [*n* = 40 ADHD, 27 male; 30 typically developing (TD), 16 male; all right-handed, 7–12 years]. The NYU institutional review board approved all procedures for data collection and sharing and written informed consent was obtained from each participant.

**Table 1:** Participant demographics.

	***n***	**Mean age (*SD*)**	**Mean full-scale IQ (*SD*)**	**Mean FD (*SD*)**	**Mean volumes < 0.5 mm FD (*SD*)**
ADHD	40	9.91 (1.24)	109.43 (14.81)	0.130 (0.042)	164.10 (7.44)
TD	30	9.43 (1.40)	112.17 (16.22)	0.129 (0.039)	163.73 (6.03)
*p*-value		0.13	0.46	0.94	0.83

In the ADHD group, 27 had an ADHD-combined diagnosis while 13 had an ADHD-inattentive diagnosis. No participants in the control group were on medication, four participants in the ADHD group were on medication, and medication status for 25 ADHD participants was not available. Two participants in the control group had a secondary diagnosis of anxiety. In the ADHD group, secondary diagnoses consisted of anxiety (3), oppositional defiant disorder (3), depression (2), reading disorder (1), phobia (1), pervasive developmental disorder (1), and dysgraphia (1).

Inclusion as a TD child was based on the absence of any current Axis-I psychiatric diagnoses as determined by administering the Kiddie-Schedule for Affective Disorder and Schizophrenia-Present and Lifetime Version (KSADS-PL) to each child and their parent. Inclusion as a child with ADHD required a clinical ADHD diagnosis based on each parent and child's responses to the KSADS-PL. IQ was evaluated using the Wechsler Abbreviated Scale of Intelligence (WASI). Finally, no psycho-stimulant drugs were administered to participants for at least 24 h prior to scanning. Full participant details can be found at http://fcon_1000.projects.nitrc.org/indi/adhd200/.

### Data acquisition

All subjects were scanned using a 3T Allegra following the diagnostic assessment. The resting state fMRI data were collected using an echo-planner imaging (EPI) sequence (*TR* = 2,000 ms; *TE* = 15 ms; flip angle = 90°; FOV = 240 mm; voxel size = 3 × 3 × 4 mm; number of slices = 33, 4 mm slice thickness; 180 volumes). Participants were asked to remain still, close their eyes, think of nothing in particular and not to fall asleep, while a black screen was presented to them.

One high-resolution T1-weighted anatomical image was acquired using a magnetization prepared gradient echo sequence (MP-RAGE: *TR* = 2,530 ms; *TE* = 3.25 ms; flip angle = 7°; FOV = 256 mm; voxel size = 1.3 × 1 × 1.3 mm; number of slices = 128, 1.33 mm slice thickness; 8.07 min). Each image was defaced to ensure patient confidentiality.

### Data preprocessing

The data were preprocessed using the Data Processing Assistant for Resting-state fMRI Advanced Edition (DPARSF-A) toolbox (Yan and Zang, 2010), along with FSL and AFNI tools. Preprocessing steps consisted of the removal of the first 5 volumes to allow for BOLD signal stabilization, slice-timing correction, realignment, normalization to the SPM EPI template (3 mm), and smoothing (6 mm FWHM using FSL). ICA-FIX denoising (Salimi-Khorshidi et al., 2014) was then applied to all subjects by first classifying noise components from 10 randomly chosen individuals with ADHD and 10 randomly chosen individuals without ADHD. ICA-FIX was then applied to create a training file of independent component noise features that was then used to regress out noise components from the data of all subjects. Noise-regression (Friston 24 motion parameters) was not used at this stage, as it was applied independently in later preprocessing steps. Previous research has shown that ICA denoising decreases non-neuronal sources of BOLD signal variability (Ciric et al., 2017) while increasing effect sizes of between-group brain signal variability differences (Garrett et al., 2010). Further preprocessing steps included despiking (AFNI's “new” 3dDespike algorithm) and nuisance signal regression Friston 24 motion parameters, cerebral spinal fluid (CSF), white matter (WM), and linear detrending using DPABI (Yan et al., 2016). Nuisance signals for WM and CSF were acquired using masks from segmented T1 brain-extracted images in SPM. The data were then band-pass filtered to isolate low frequency fluctuations that characterize resting-state BOLD signals (0.01–0.1 Hz). Additional supplementary analyses were conducted without the use of WM and CSF nuisance regressors.

### Calculation of voxel-wise MSSD

Preprocessed resting-state fMRI data were first normalized to z-statistics before calculation of voxel-wise MSSD statistics for each subject (Samanez-Larkin et al., 2010; Nomi et al., 2017). MSSD is calculated by subtracting time point t from time point t+1 then squaring the result. The squared values across the entire time series are then averaged together to produce a single MSSD metric for each voxel for each subject (Von Neumann et al., 1941).

$$\begin{array}{l} \delta^{2}=\;\frac{\sum_{i\;=\;1}^{n-1}{\left(x_{i+1}-\;x_{i}\right)}^{2}}{n-1} \end{array}$$

### Categorical and dimensional analyses

All analyses were carried out on subject-level whole-brain voxel-wise MSSD maps using ordinary least squares (OLS) regression in FSL (Benjamini and Hochberg, 1995). A categorical analysis compared MSSD values between children with an ADHD diagnosis and children without an ADHD diagnosis (i.e., TD children). Dimensional analyses examined the relationship between voxel-wise whole-brain MSSD and ADHD symptom severity across all children regardless of ADHD diagnosis. Three measures of symptom severity were examined in the current study from the Conners' Parent Rating Scales-Revised: Long-version (CPRS-LV). They were the ADHD-index (identifies children “at risk” for ADHD), ADHD-inattentive (correspondence with the DSM-IV diagnostic criteria for inattentive type of ADHD), and ADHD-hyperactive (correspondence with the DSM-IV diagnostic criteria for hyperactive type of ADHD) scores. One participant with an ADHD diagnosis and one participant without an ADHD diagnosis did not have behavioral scores for all three measures; these participants were excluded from all dimensional analyses. Both categorical and dimensional analyses included handedness, gender, and FD as nuisance regressors in the OLS model. All analyses were carried out using *p* < 0.05 for voxel-wise (uncorrected) and *p* < 0.05 for cluster-wise (Guassian Random Field theory corrected) significance thresholds.

## Results

### Categorical analysis

There were no significant voxel-wise differences surviving cluster-correction between children with ADHD and TD children across the whole brain.

### Dimensional analyses

**Figure 1:**
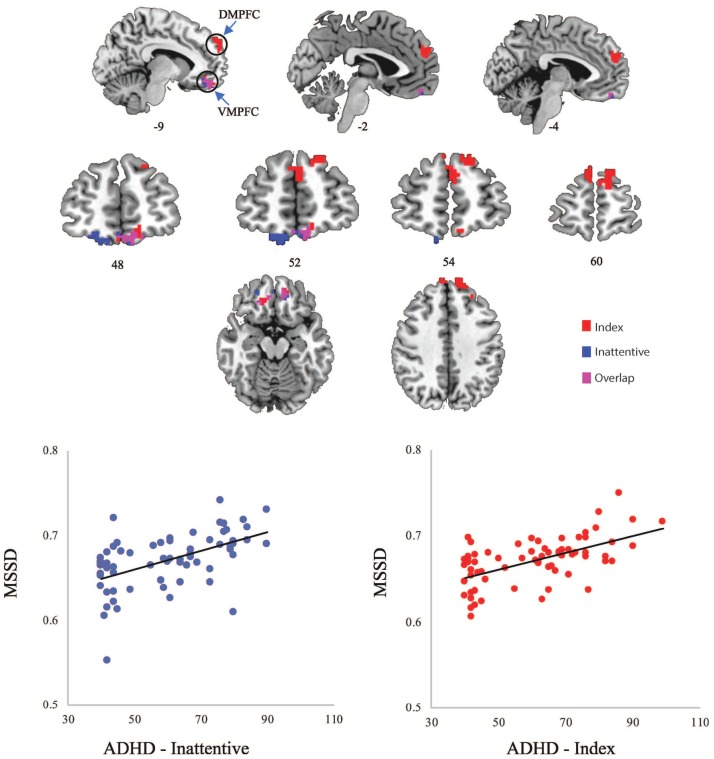
**(Top)** Significant clusters of MSSD values related to ADHD-Index (red) and ADHD-Inattentive (blue) scores; **(Bottom)** Scatterplots of averaged MSSD values within clusters plotted against ADHD scores. DMPFC, dorsal medial prefrontal cortex; VMPFC, ventral medial prefrontal cortex.

Significant relationships with MSSD were found for ADHD-index and ADHD-inattentive symptom severity scores across children with and without an ADHD diagnosis (Figure 1). The cluster-corrected results demonstrated that higher scores (e.g., increased symptom severity) on the ADHD-index measure were related to increased MSSD in the dorsal MPFC and ventral MPFC, while higher scores on the ADHD-inattentive measure were associated with greater MSSD in the ventral MPFC. No significant relationships between MSSD and ADHD-hyperactive scores were observed.

Additional analyses were conducted without the use of WM and CSF nuisance regressors. The average within-subject correlation between the MSSD values without WM and CSF nuisance regressors and MSSD values with WM and CSF nuisance regression were extremely high (*r* = 0.997, *SD* = 0.0017), demonstrating the preservation of spatial MSSD patterns between the two preprocessing pipelines. Similar spatial representations of positive correlations between MSSD and ADHD symptomatology were also observed (Supplementary Figure 1).

*Post-hoc* Spearman's rho correlations were conducted in order to demonstrate that the positive relationship between MSSD values and ADHD symptomatology remained significant when averaging MSSD values across voxels within identified clusters. Significant correlations were found between average MSSD values within the MPFC and ADHD-index scores (rho = 0.502, *p* = 0.000013) and also between average MSSD values within the ventral MPFC and ADHD-inattentive scores (rho = 0.506, *p* = 0.000011).

### Framewise displacement and MSSD

In order to examine the influence of head motion on brain signal variability, an additional OLS regression was run using mean FD values to predict MSSD values. The results showed both positive and negative relationships between FD and MSSD across various areas of the cortex (Supplementary Figure 2) demonstrating the importance of controlling for head motion in brain signal variability studies.

## Discussion

The current study provides initial evidence that voxel-wise intrinsic brain signal variability is related to ADHD symptom severity. This relationship was only present in a dimensional analysis examining ADHD symptom severity across children with and without a clinical diagnosis of ADHD. There were no categorical differences in voxel-wise whole-brain brain signal variability between children with and without a diagnosis of ADHD. The examination of dimensional relationships between brain signal variability and ADHD symptomatology regardless of ADHD diagnosis follows recent efforts that focus on quantitative measures of clinical disorders [e.g., Research Domain Criteria (RDoC), (Insel et al., 2010)] rather than categorical separations of diagnosis/no diagnosis (Uddin et al., 2017).

The current results also demonstrate that increased resting-state brain signal variability may underlie increased task-based brain signal variability in individuals with ADHD (Depue et al., 2010; Sørensen et al., 2016). This builds on previous work showing that the brain can have a similar functional organization during rest and task (Cole et al., 2014; Bolt et al., 2017) and that functional properties of the brain at rest can predict functional properties of the brain during task (Cole et al., 2016; Tavor et al., 2016). Thus, increased task-based brain signal variability in ADHD may not be solely a result of task-driven influences. The current study suggests that increased task-based pre-frontal cortex brain signal variability related to ADHD symptomatology may be partly driven by endogenous rather than exogenous factors.

The current finding of a relationship between brain signal variability in the dorsal and ventral MPFC and ADHD symptom severity is in line with previous task-based and resting-state fMRI studies identifying aberrant MPFC brain activity in ADHD. Previous task-based fMRI studies in adolescents and adults demonstrated categorical effects where individuals with ADHD showed increased brain signal variability in the MPFC compared with individuals without ADHD (Depue et al., 2010; Sørensen et al., 2016). A previous resting-state fMRI study in children showed atypical functional connections underlying both categorical and dimensional aspects of ADHD in the dorsal MPFC and ventral MPFC (Elton et al., 2014). Finally, decreased regional homogeneity (functional connections between neighboring voxels) in the PFC in individuals with ADHD has been found in previous resting-state fMRI studies in children (Cheng et al., 2012; An et al., 2013) and adults (Uddin et al., 2008; Liu et al., 2010). The current study builds on these previous studies by demonstrating that resting-state brain signal variability within the MPFC is related to ADHD symptom severity in children.

The dorsal and ventral MPFC are anterior nodes within the default mode network (DMN) (Andrews-Hanna et al., 2010). The DMN is generally involved in internally oriented, evaluative cognitive processes (Uddin et al., 2007). More specifically, the dorsal MPFC has been implicated in a DMN subsystem that is related to self-relevant and affective processing, while the ventral MPFC has been implicated in a DMN subsystem tied to mental imagery (Andrews-Hanna et al., 2010). Atypical activation in posterior nodes of the DMN such as the precuneus has been identified in ADHD in a task-based meta-analysis (Cortese et al., 2012) while several resting-state studies have found decreased functional connections between anterior and posterior nodes of the DMN (Castellanos and Aoki, 2016). In tasks engaging executive function processes, the DMN typically deactivates while an externally-oriented central executive network (CEN) increases in activation. Increased anti-correlations between the DMN and CEN during rest and task have been tied to reduced reaction time variability in a flanker task in typical individuals (Kelly et al., 2008). Increased reaction time variability commonly found in individuals with ADHD may be accounted for by atypical relationships between the DMN and CEN, with DMN dysfunction playing a role in DMN-CEN anti-correlation strength (Castellanos and Proal, 2012). Underlying differences in brain signal variability of network nodes within the DMN during rest may contribute to atypical DMN-CEN dysfunction during task, and in turn may help to explain attentional lapses in ADHD that lead to inefficient cognitive processing.

The utility of brain signal variability measures for providing meaningful information about brain activity apart from measures of BOLD activation strength has been demonstrated in previous research. Depue and colleagues showed that brain areas differing in brain signal variability between individuals with and without a diagnosis of ADHD did not differ in univariate activation strength, while brain areas that did differ in univariate activation strength between groups did not differ in brain signal variability (Depue et al., 2010). Garret and colleagues showed that differences in brain signal variability (measured by standard deviation) during fixation periods between visual detection, perceptual matching, attentional cueing, and working memory trials predicted age better than differences in the mean level of BOLD activation (Garrett et al., 2010). These studies demonstrate the utility of brain signal variability for providing unique information that is at times unrelated to BOLD activation measures. Thus, the findings of the current study provide another avenue to describe atypical BOLD signal properties related to ADHD symptomatology that can offer unique information compared with measures of functional connectivity or univariate BOLD activation levels.

The finding of a positive relationship between increased brain signal variability and ADHD symptomatology in the current study during rest and previous studies during task (Depue et al., 2010; Sørensen et al., 2016) is seemingly at odds with the plethora of research demonstrating that increased brain signal variability is associated with younger individuals (Nomi et al., 2017) and improved task performance (Garrett et al., 2013). However, optimal brain function may be supported by a Yerkes-Dodson curve where increased brain signal variability becomes detrimental to cognitive performance after a yet unidentified critical window. It may be the case that individuals with ADHD have brain areas within the PFC that surpass this optimal window of variability, where it becomes detrimental to their cognitive performance. Additional considerations should also be given to differences in the type of paradigm (rest vs. task), the measurement of brain signal variability (e.g., standard deviation vs. MSSD), and differences in individual study population samples. Further research is needed to clarify the relationship between brain signal variability, cognition, and clinical symptomatology.

Finally, brain signal variability has also become an important avenue of investigation in autism spectrum disorder (ASD), with researchers proposing that atypical variability may have cognitive and behavioral consequences for afflicted individuals (Dinstein et al., 2015). Functional connectivity studies have recently demonstrated that hyper-variability may be responsible for the hypo-connectivity between brain areas often present in ASD (Falahpour et al., 2016; Chen et al., 2017). Because individuals with ASD are often co-diagnosed with ADHD (Simonoff et al., 2008), brain signal variability is increasingly becoming an important area of investigation for both ASD and ADHD. Investigating the relationship between brain signal variability, ASD, and ADHD would provide valuable insight into the nature of each disorder, and the consequences of comorbidity for cognition and behavior (Dajani et al., 2016).

### Limitations

One limitation of the current study may be related to the significance threshold (*p* < 0.05) for voxel-wise correction. Previous research on task-based fMRI data suggests that a stricter voxel-wise criterion (*p* < 0.001) in conjunction with a cluster-wise criterion (*p* < 0.05) provides better protection against type I errors (Eklund et al., 2016). However, as previous work focused on false positives related to task-based fMRI activation clusters, it is currently unknown how this translates to investigations of resting-state brain signal variability.

Another concern is the use of participants with both a combined diagnosis and inattentive diagnosis of ADHD. Previous research has shown that differences in functional connections can be observed between ADHD-combined and ADHD-inattentive diagnostic groups in fMRI research (Fair et al., 2012). The current study used a combination of both diagnostic groups, as the main concern was to equate ADHD and control groups on IQ and head motion. As head motion has been shown to artificially increase the magnitude of the BOLD signal (Power et al., 2012), it may be possible that increased head motion can lead to increased BOLD signal variability. A supplementary analysis using FD as a predictor of MSSD in an OLS voxel-wise regression analysis showed both positive and negative relationships between FD and MSSD. We note that both groups had extremely low FD due to careful participant selection and matching criteria. This demonstrates the importance of controlling for head motion in brain signal variability studies (Supplementary Figure 2). Thus, the current study prioritized avoiding group differences in head motion between children with and without an ADHD diagnosis. Future work should investigate associations between ADHD subtypes and brain signal variability.

## Conclusions

The current study demonstrates that resting-state BOLD signal variability is related to dimensional, but not categorical differences in ADHD symptomatology. The dimensional analysis demonstrated that areas within the medial PFC are positively correlated with measures of the ADHD index and ADHD inattentive symptom severity. These results provide initial evidence that resting-state brain signal variability in children is a viable avenue of investigation to identify brain function related to ADHD symptomatology.

## Author contributions

JN, AH, and LQU designed the study. JN, ES, WV, and TB analyzed the data. JN and AH contributed unpublished analytic materials. JN, ES, WV, TB, AH, and LQU wrote the manuscript and approved the final version.

### Conflict of interest statement

The authors declare that the research was conducted in the absence of any commercial or financial relationships that could be construed as a potential conflict of interest.
