# Corrigendum: Resting-State Brain Signal Variability in Prefrontal Cortex Is Associated With ADHD Symptom Severity in Children

**DOI:** 10.3389/fnhum.2019.00431

**Published:** 2019-12-18

**Authors:** Jason S. Nomi, Elana Schettini, Willa Voorhies, Taylor S. Bolt, Aaron S. Heller, Lucina Q. Uddin

**Affiliations:** ^1^Department of Psychology, University of Miami, Coral Gables, FL, United States; ^2^Department of Psychiatry and Human Behavior, Brown University, Providence, RI, United States; ^3^Neuroscience Program, University of Miami Miller School of Medicine, Miami, FL, United States

**Keywords:** ADHD, brain signal variability, resting-state fMRI, MSSD, prefrontal cortex

In the original article, there was a mistake in Figure 1, Supplementary Figure 1, and Supplementary Figure 2 as published. The figures use the measure “MSSD^2^” (MSSD squared) instead of “MSSD.” The corrected Figure 1, Supplementary Figure 1, and Supplementary Figure 2 appear below.

**Figure 1:**
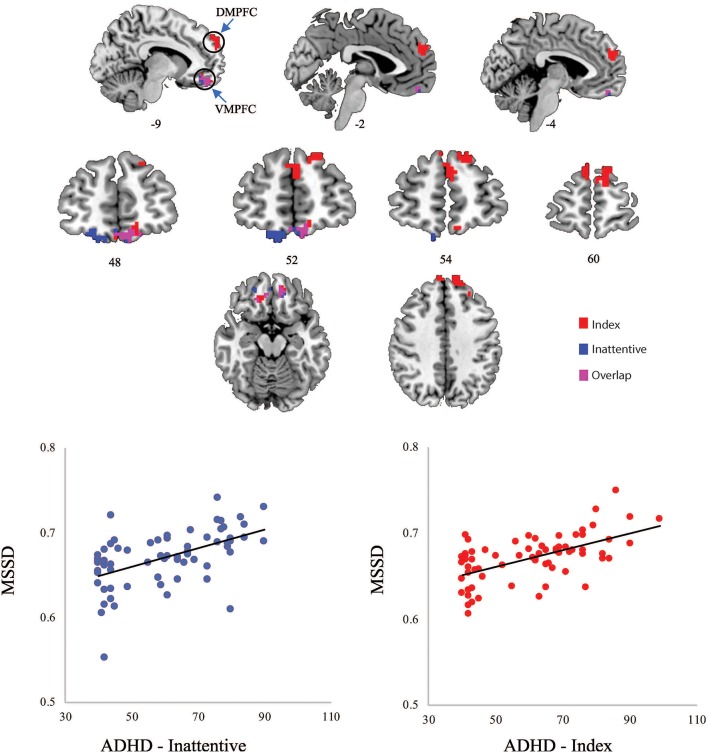
**(Top)** Significant clusters of MSSD values related to ADHD-Index (red) and ADHD-Inattentive (blue) scores; **(Bottom)** Scatterplots of averaged MSSD values within clusters plotted against ADHD scores. DMPFC, dorsal medial prefrontal cortex; VMPFC, ventral medial prefrontal cortex.

**Supplementary Figure 1:**
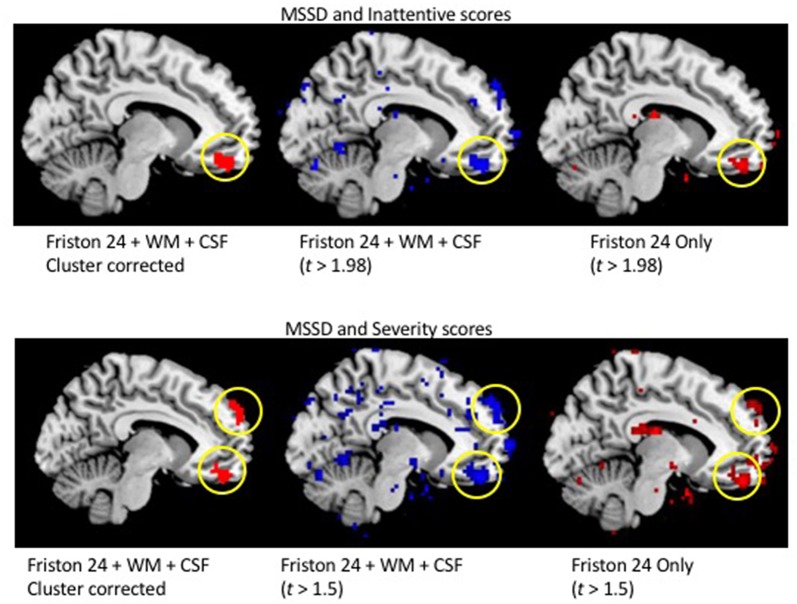
Similar spatial patterns of the relationship between MSSD and ADHD behavioral scores with (center) and without (right) white matter (WM) and cerebral spinal fluid (CSF) nuisance regression.

**Supplementary Figure 2:**
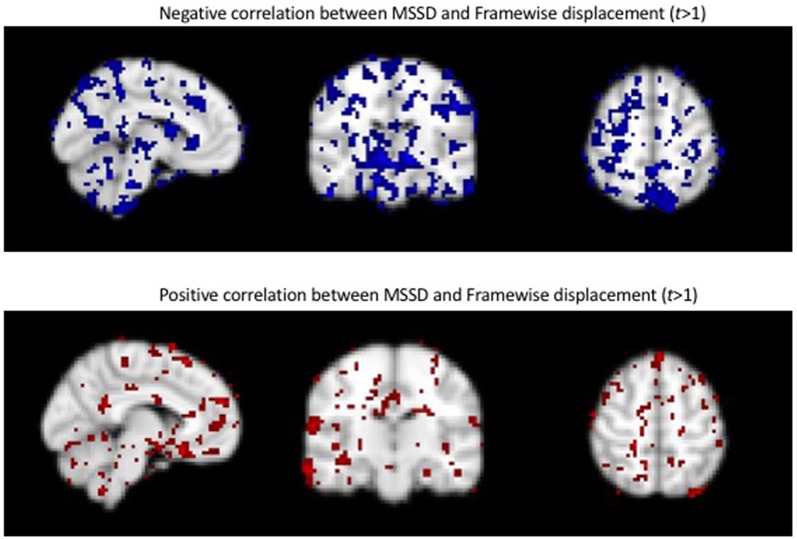
Positive and negative relationships between MSSD and framewise displacement (FD).

Additionally, the analysis discussed in the article also uses “MSSD^2^” rather than “MSSD.”

A correction has been made to the **Results**, subsection **Dimensional Analyses**, paragraphs one, two, and three:

“Significant relationships with MSSD were found for ADHD-index and ADHD-inattentive symptom severity scores across children with and without an ADHD diagnosis (Figure 1). The cluster-corrected results demonstrated that higher scores (e.g., increased symptom severity) on the ADHD-index measure were related to increased MSSD in the dorsal MPFC and ventral MPFC, while higher scores on the ADHD-inattentive measure were associated with greater MSSD in the ventral MPFC. No significant relationships between MSSD and ADHD-hyperactive scores were observed.”

“Additional analyses were conducted without the use of WM and CSF nuisance regressors. The average within-subject correlation between the MSSD values without WM and CSF nuisance regressors and MSSD values with WM and CSF nuisance regression were extremely high (*r* = 0.997, *SD* = 0.0017), demonstrating the preservation of spatial MSSD patterns between the two preprocessing pipelines. Similar spatial representations of positive correlations between MSSD and ADHD symptomatology were also observed (Supplementary Figure 1).”

“*Post-hoc* Spearman's rho correlations were conducted in order to demonstrate that the positive relationship between MSSD values and ADHD symptomatology remained significant when averaging MSSD values across voxels within identified clusters. Significant correlations were found between average MSSD values within the MPFC and ADHD-index scores (rho = 0.502, *p* = 0.000013) and also between average MSSD values within the ventral MPFC and ADHD-inattentive scores (rho = 0.506, *p* = 0.000011).”

The authors apologize for this error and state that this does not change the scientific conclusions of the article in any way. The original article has been updated.

