# Supplementary material for: Resting-State Brain Signal Variability in Prefrontal Cortex Is Associated With ADHD Symptom Severity in Children
Source: Front Hum Neurosci. 2018 Mar 12;12:90. doi: 10.3389/fnhum.2018.00090 (PMC5857584; doi:10.3389/fnhum.2018.00090)
Supplement: Supplementary file 1 [file DataSheet1.DOCX]

**Supplementary Materials**

**Supplementary Figure 1**: Similar spatial patterns of the relationship between MSSD and ADHD behavioral scores with (center) and without (right) white matter (WM) and cerebral spinal fluid (CSF) nuisance regression.

**Supplementary Figure 2**: Positive and negative relationships between MSSD and framewise displacement (FD).
